# Saliency-Based Bleeding Localization for Wireless Capsule Endoscopy Diagnosis

**DOI:** 10.1155/2017/8147632

**Published:** 2017-11-28

**Authors:** Hongda Chen, Shaoze Wang, Yong Ding, Dahong Qian

**Affiliations:** ^1^The Institute of VLSI Design, Zhejiang University, Hangzhou, China; ^2^Faculty of School of Medicine, Zhejiang University, Hangzhou, China

## Abstract

Stomach bleeding is a kind of gastrointestinal disease which can be diagnosed noninvasively by wireless capsule endoscopy (WCE). However, it requires much time for physicians to scan large amount of WCE images. Alternatively, computer-assisted bleeding localization systems are developed where color, edge, and intensity features are defined to distinguish lesions from normal tissues. This paper proposes a saliency-based localization system where three saliency maps are computed: phase congruency-based edge saliency map derived from Log-Gabor filter bands, intensity histogram-guided intensity saliency map, and red proportion-based saliency map. Fusing the three maps together, the proposed system can detect bleeding regions by thresholding the fused saliency map. Results demonstrate the accuracy of 98.97% for our system to mark bleeding regions.

## 1. Introduction

Bleeding is one of the most common lesions about gastrointestinal (GI) disease [1]. The conventional detection method requires physicians to scan real-time images through an endoscope-attached wire, which is clinically invasive. Capsule endoscope camera, on the distal side of which a miniaturized sensor is mounted, can sweep past patients' gut wall. Such noninvasive endoscopy, called wireless capsule endoscopy (WCE), can capture a video of nearly 57,000 frames, where the bleeding locations are recorded. Detecting and interpreting the lesions from numerous frames need physicians to be concentrated [2]. To ease the task of subjective diagnosis, computer-assisted bleeding localization systems are proposed [3].

The state-of-the-art bleeding localization systems depend mainly on color features. For instance, authors in [4] extracted mean and variance in the HSI color space: hue, saturation, and intensity. In [5], high-order statistical moments including kurtosis were added to the RGB color space. Similarly, statistical features based on intensity histogram [6] and local binary pattern are computed in the RGB and HSV color space. Unlike color features, edge was discarded since it increased false positive (FP) rate [7]. More importantly, these systems generally compose these statistical features from different color spaces into a vector, followed by a supervised pattern classification tool such as artificial neural network or support vector machine. As a result, the task of localization is degraded since classification fails to predict accurate positions of a lesion.

This paper proposes a saliency detection method that is devoted to localizing the bleeding regions in WCE images. Visual saliency models that extract the most interesting part from an image have been proposed in the nature image recognitions [8–11]. However, it is undesirable to apply the models directly to medical image analysis. Firstly, most existing saliency models assume that the visual fixation tends to stay within the center of images [12], while, in medical images, the fixation mainly refers to lesions which appear anywhere in the field of view. Secondly, salient regions in the natural images tend to be in the foreground where edge and color information is quite distinguishable; however, lesions in the WCE images vary according to the specific diseases. For instance, the bleeding lesions are small and dark red, while the colon lesions appear large and highly laminated. Thirdly, in the bleeding localization system, the salient region must exclude not only normal tissue, but also gastric fluid and undigested residue. The latter two are quite different from the normal, which can prevent the system from recognizing FP samples.

To detect the bleeding regions with both high sensitivity and specificity, the traditional methods take full advantage of color features and ignore other features. However, when diagnosing the bleeding area, physicians use multiple features, such as edge, intensity, and color. In order to simulate the diagnosis process, a visual saliency-based WCE bleeding detection system is proposed, which includes three kinds of saliency maps, color, intensity, and edge. The main contribution of this paper is that intensity and edge saliency maps are introduced to better imitate the diagnosis of physician. Salient intensity is extracted from the intensity histograms which have rotation invariance while edges are selected based on the phase congruency [9] of Log-Gabor filter bank which can solve the problem of nonuniform illumination of the WCE. Experimental results demonstrate that our algorithm has a very good performance for detecting the bleeding area. The rest of this paper is organized as follows. Materials and methods are presented in Section 2; Section 3 exhibits the experimental results; finally, some conclusion is drawn in Section 4.

## 2. Materials

**Figure 1:**
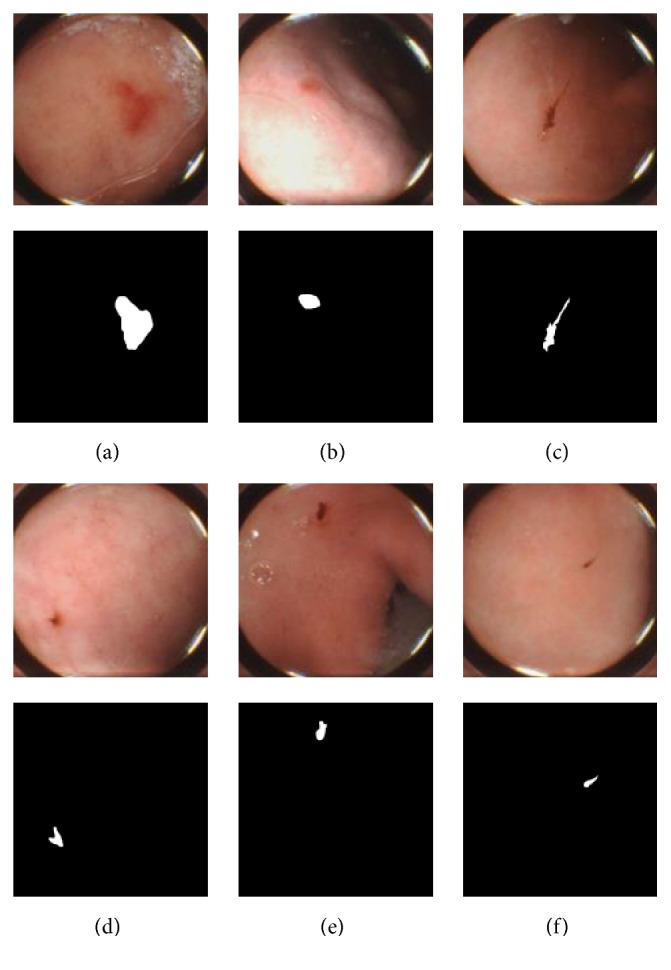
Bleeding images and masks.

A total of 200 WCE frames, with the pixel resolution of 480-by-480, are used with a warrant from Ankon Incorporation, Wuhan, China. Two physicians, with three-year clinical experience, drew bleeding masks independently; the two masks of each image are fused together by pixel-to-pixel AND operation. Randomly selected six bleeding images and masks are presented in Figure 1.

## 3. Saliency Map Extraction

Clinicians discriminate the bleeding lesions from a WCE image mainly based on some salient information. Here we classify the information into three categories: edge information, intensity information, and color information, each of which is quantified using a saliency map.

### 3.1. Edge Saliency Map

When clinicians calibrate the bleeding location, the clear contour of bleeding area is an important reference condition. Therefore, the outlines of bleeding area will occupy a large proportion in the clinician's attention mechanism to distinguish the bleeding area from the normal. In order to simulate the diagnosis of clinician, we propose the edge saliency map.

Generally, edges can be obtained from spatial edge detectors such as Roberts, Laplace, or Canny, but all these detectors are vulnerable to the image noise. Edge detection based on PC has the brightness invariance properties, which can compensate for the instability luminance of color channels [13]. The solution of extracting noise-insensitive edge is to convert an image into the spectrum domain and to calculate the phase congruency (PC [9]). Still, there are many frequency transformation tools like Fast Fourier Transformation (FFT), discrete cosine transformation (DCT), and Gabor filters. However, the FFT and DCT fail to be analyzed in the multiresolution of scales and orientations, which make the PC too coarse. Though Gabor filters can be used in the different scales and orientation, they tend to be bias frequency components towards the lowest band. Hence, the PCs calculated from Gabor frequency bands are not isolated. Here we choose the Log-Gabor wavelet transform because it is competent to extract the PC at the isolated center frequencies with symmetry attenuation responses [14]. The scales and orientations parameters are empirically set to 5 and 6, respectively. According to the spectrum analysis, an image can be decomposed into a combination of amplitude spectrum and phase spectrum. Accordingly, the Log-Gabor filter is constructed by multiplying the frequency response of the two components together in the polar coordinates system as follows:

(1) $$\begin{array}{c} LG\left(\;\rho ,\theta \;\right)=\exp\left\{\;-\frac{\log\left(\rho /f_{0}\right)}{2{\sigma_{\rho }}^{2}}\;\right\}\cdot \exp\left\{\;-\frac{{\left(\theta -\theta_{0}\right)}^{2}}{2{\sigma_{\theta }}^{2}}\;\right\}, \end{array}$$

where (*ρ*, *θ*) represents the polar coordinates and *f*_0_ is the center frequency of the filter and it is related to our current scale *n* by *f*_0_ = minWave × mult^*n*^ in which minWave is the wavelength of smallest scale filter and mult is the scaling factor between the successive filters. *θ*_0_ is the orientation angle of the filter and *σ*_*ρ*_ and *σ*_*θ*_ determine the scale bandwidth and the angular bandwidth, respectively. In our experiments, the parameters for the Log-Gabor filters are set as follows: minWave = 6.0, mult = 2, *σ*_*ρ*_ = 0.75,  *σ*_*θ*_ = 0.6.

At each orientation, a PC map in the *n*th scales is fused by

(2) PCx,y=∑nIx,y∗Rn2+∑nIx,y∗Mn2ε+∑nIx,y∗Rn2+Ix,y∗Mn2,

where *R*_*n*_ and *M*_*n*_ denote the even and odd spectrums at the *n*th scale. The constant *ε* stabilizes the denominator. The *ε* value of 0.01 is used for all the results presented in this paper.

**Figure 2:**
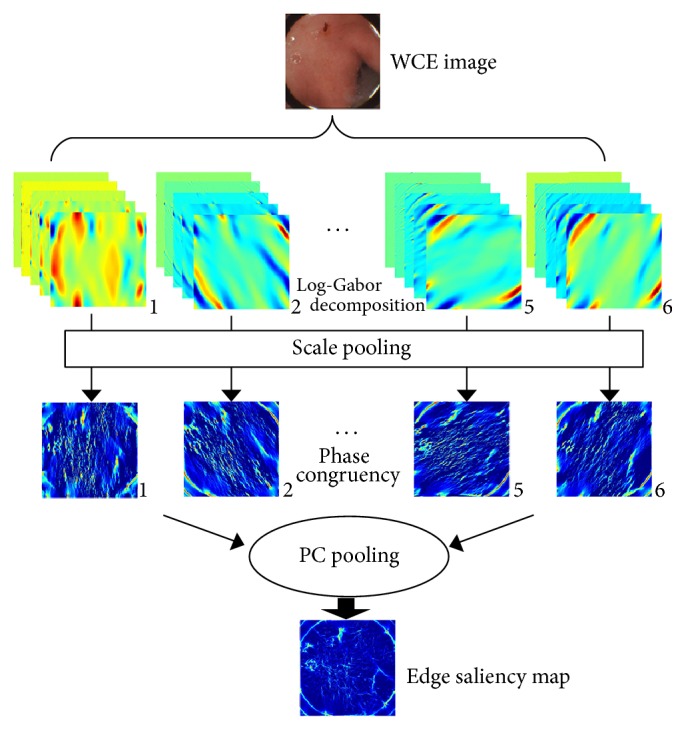
The flowchart of extracting edge saliency map.

Now five interscale PC maps at each orientation are fused together. The edge saliency map is obtained by maximizing the singular value-based moment of PC maps at six orientations:

(3) Cxx,y=∑dPCx,y×sin⁡θ2,Cyx,y=∑dPCx,y×cos⁡θ2,Cxyx,y=∑dPCx,ycos⁡θ×PCx,ysin⁡θ,S1x,y=Cxx,y+Cyx,y+Cxyx,y2Cxx,y−Cyx,y2,

where *θ* denotes the orientation angle. *S*_1_(*x*) is the edge saliency map.  Figure 2 provides the flowchart of extracting the PC-based edge saliency map, and Figure 4(b) visualizes the final high-resolution edge saliency maps.

### 3.2. Intensity Saliency Map

**Figure 3:**
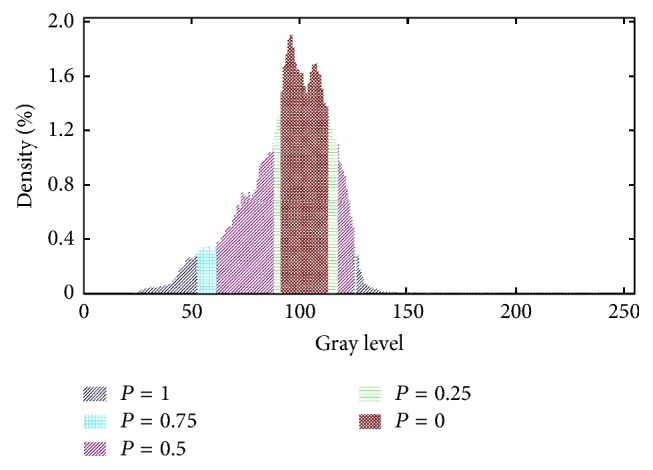
The histogram of a gray-scale WCE image where different probabilistic regions are marked with different colors.

**Figure 4:**
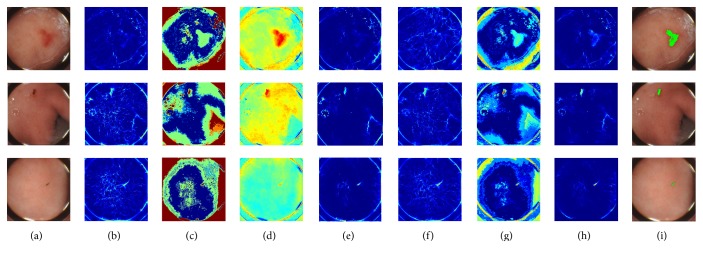
The WCE images with the bleeding and the corresponding different saliency maps. (a) Original WCE images. (b) Edge saliency map. (c) Intensity saliency map. (d) Color saliency map. (e) Fusion of the edge and intensity saliency map. (f) Fusion of the edge and color saliency map. (g) Fusion of the intensity and color saliency map. (h) Fusion of the three saliency map. (i) Suspected bleeding regions (green) by thresholding the fused saliency map.

The PC-based edge saliency map is sensitive to bleeding contours. On the other hand, the luminance intensities inside the contours are also prone to saliency. The difference in luminance is an indispensable condition for clinicians to distinguish between normal tissue and diseased tissue. Based on this principle, the gray-scale image histogram is exploited to compute an intensity saliency map. The histogram shown in Figure 3 is built by counting the gray-scale intensities within the bins. The counts of each bin are normalized to be a probabilistic value (*p*_*i*_) such that

(4) $$\begin{array}{c} \overset{N}{\underset{k=0}{\sum }}p_{k}=1, \end{array}$$

where *k* denotes the *k*th bin. There are totally *N* bins, and, in this paper, *N* = 256, which means that one gray-scale corresponds to one bin.

(5) $$\begin{array}{c} S_{2}\left(\;x,y\;\right)=\left\{\begin{array}{cc} 0 & p_{I\left(x,y\right)}>\overset{-}{p}\\ 0.25 & \lambda \overset{-}{p}<p_{I\left(x,y\right)}\leq \overset{-}{p}\\ 0.5 & \lambda^{2}\overset{-}{p}<p_{I\left(x,y\right)}\leq \lambda \overset{-}{p}\\ 0.75 & \lambda^{3}\overset{-}{p}<p_{I\left(x,y\right)}\leq \lambda^{2}\overset{-}{p}\\ 1 & p_{I\left(x,y\right)}\leq \lambda^{3}\overset{-}{p}, \end{array}\right. \end{array}$$

where $\overset{-}{p}$ and *λ* are set to 1/72 and 0.6, empirically; (*x*, *y*) denotes the pixel position in the gray-scale image *I*. The function values constitute the intensity saliency map, which is visualized in Figure 4(c).

### 3.3. Color Saliency Map

Besides the above two features, the color feature which is the most widely used in the bleeding area detection cannot be ignored. The obvious color information of the bleeding area is the most intuitive reference for the clinicians, so the state-of-the-art methods utilize the color feature to distinguish the bleeding from other regions [5, 15–17]. In perspective of the visual saliency, color is another visual stimulus that is isotropic to the contour and luminance. Hence color saliency map is also computed and fused together with the above two maps. As most bleeding lesions appear to be red, we calculate the proportion of the red of the color saliency map,

(6) $$\begin{array}{c} S_{3}\left(\;x,y\;\right)=\frac{r\left(x,y\right)}{r\left(x,y\right)+g\left(x,y\right)+b\left(x,y\right)+\varepsilon }, \end{array}$$

where [*r*, *g*, *b*] is a color vector at the pixel position of (*x*, *y*) and *ε* is a small constant for the purpose of stabilization.

The aforementioned three saliency maps localize the bleeding ROIs in WCE images from different aspects. More importantly, the quantities in all the three saliency maps are between 0 and 1; thus we can derive the fused saliency map by fusing them together by the following formula, and the three saliency maps are pooled by

(7) $$\begin{array}{c} S\left(\;x,y\;\right)=\frac{1}{3}\overset{3}{\underset{i=1}{\sum }}S_{i}\left(\;x,y\;\right). \end{array}$$

As an illustration, Figure 4 presents the three test WCE images in Figure 4(a). For each image, three isolated saliency maps and the contrastive fused saliency maps are arranged from Figures 4(b)–4(h).  Figure 4(i) localizes the suspected bleeding ROIs by thresholding the fused saliency map.

## 4. Experimental Results and Discussion

To evaluate our saliency-based bleeding detection system, we compared the saliency maps with the golden-standard masks that are marked by two physicians. As each saliency map implies the probability of bleeding positions, a receiver operator characteristic (ROC) curve can illustrate both sensitivity and specificity of a map. Both criteria are defined as follows:

(8) Sensitivity=TPTP+FN,Specificity=TNFP+TN,

where TP denotes true positive rates and FP false positive rates; TN and FN denote true negative and false negative, respectively. TP means that a bleeding pixel is correctly classified by a saliency map while FP means that a nonbleeding pixel is incorrectly regarded as bleeding.

### 4.1. Quantitative Analysis

**Figure 5:**
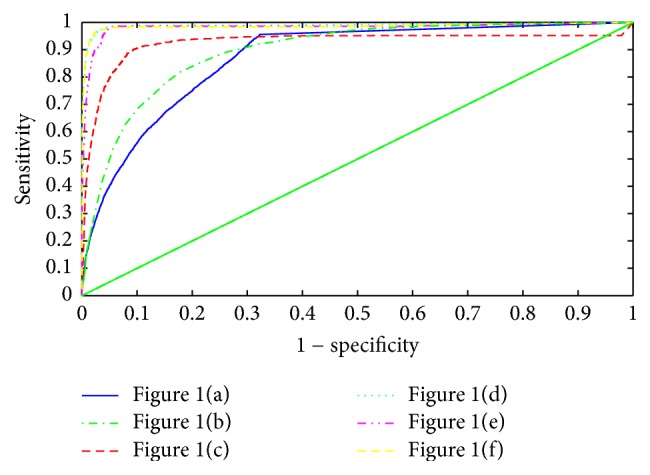
ROC curves of fused saliency maps with regard to six images.

Given that a threshold increased from 0 to 1, a series of sensitivity and specificity values are obtained. Relying on these values, a ROC curve can be plotted. As an illustration, Figure 5 plots the ROC curves of fused saliency maps with regard to the six images in Figure 1.

From Figure 5, it can be observed that the fused saliency map shows unbalanced performance on the six representative bleeding images. Area under curves (AUC) is 0.994, 0.989, 0.986, 0.926, 0.895, and 0.869, respectively. Note that the fused saliency map tends to be more sensitive to tiny and sharp bleeding region (Figure 1(f)) than opaque one (Figure 1(a)). This may result from the fusion strategy which weights three isotropic maps equivalently.

To further evaluate the performance of the proposed saliency map, the three kinds of saliency maps, saliency maps of different combinations, and the peer algorithm [17] are listed in Table 1, where accuracy, sensitivity, and specificity values for different saliency maps are presented. The accuracy criterion is computed as

(9) $$\begin{array}{c} Accuracy=\frac{TP+TN}{TP+TN+FP+FN}, \end{array}$$

where TP, TN, FP, and FN are obtained based on the threshold that is the mean value.

**Figure 6:**
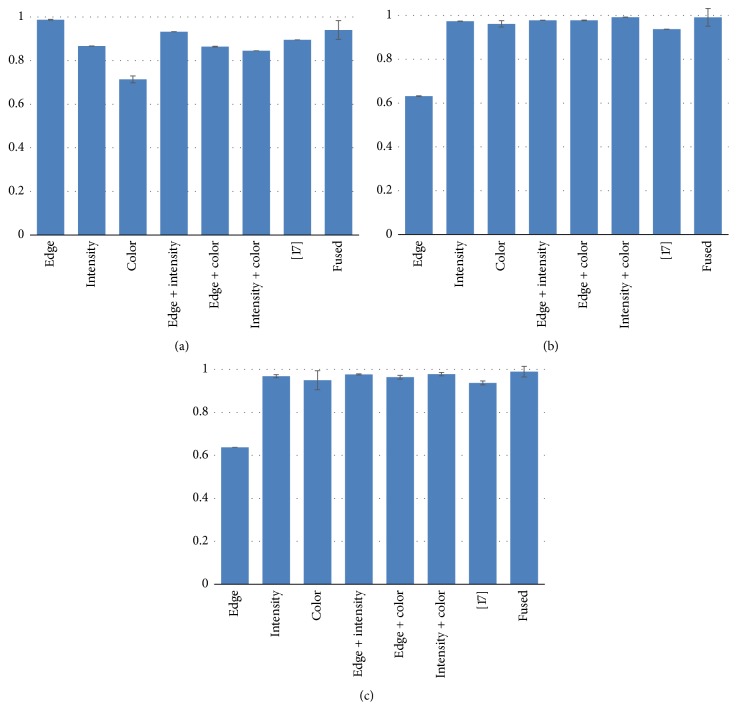
(a) Sensitivity of 200 WCE bleeding frames. (b) Specificity of 200 WCE bleeding frames. (c) Accuracy of 200 WCE bleeding frames.

It can be observed that edge and intensity saliency maps are helpful to enhance bleeding detection accuracy. The edge saliency map has high degree of sensitivity to improve the sensitivity of the fused saliency map and the higher specificity of the fused saliency map benefits from the intensity saliency map; it is clear that the proposed saliency map is better than the saliency map in [17], which uses single color features. In order to discuss the robustness of the proposed saliency map, the histogram is drawn in Figure 6. From Figure 6(a), it can be seen that the sensitivity of edge saliency map is better than the other saliency maps; the specificity in Figure 6(b) illustrates that effect of the fused saliency map is very ideal and the values are very close to 1, while the main contribution of specificity of the fused saliency map is from the intensity saliency map; Figure 6(c) shows that the accuracy of the fused saliency map is the best, which meets the expectations of our design.

In consideration of the impact of the color space [18–20], the fused saliency map is applied to the different color spaces in Table 2. From Table 2, the fused saliency map in the RGB color space has better performance than the HSI and the HSV.

In addition, the proposed saliency map provides the probability of bleeding, which is suitable to be used as a postprocessing step in a superpixel detection flowchart [21]. Based on the saliency map, unimportant superpixels can be discarded.

### 4.2. Qualitative Analysis

**Figure 7:**
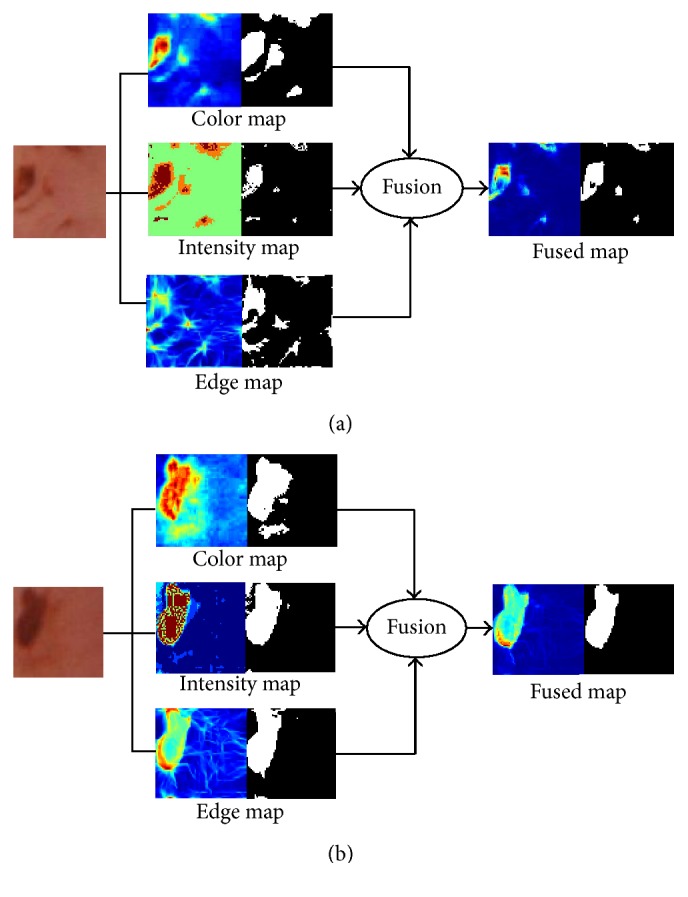
Procedure of localization for bleeding area.

In order to intuitively see the effect of the saliency map, we delineate the ROI of bleeding point. As shown in Figure 7, there is the binary image next to each saliency map. The saliency map is binarized according to single thresholding. The threshold of a saliency map is determined by the mean value of all quantities in the saliency map.

It can be seen that color binary mask is oversized while the shape binary mask is undersized. The edge binary mask tends to reveal all potential bleeding regions. Instead of applying logic AND or OR operations to fusing the three isolated binary masks, the proposed fusion strategy performs probabilistic calculation on isolated saliency maps, followed by binarizing the fused saliency map, which increases the fidelity of TP regions. It is obvious to see in Figure 7 that the fused binary map reveals exactly where the bleeding point is.

### 4.3. Image Noise Influence

**Figure 8:**
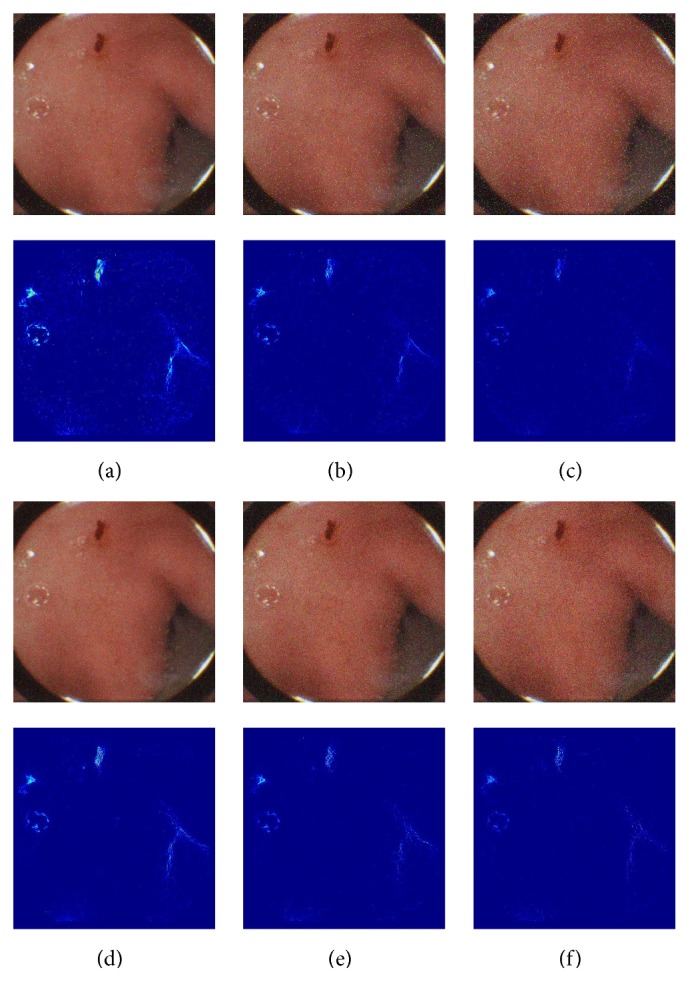
WCE image with different level noise and the fused saliency map of the image. (a), (b), and (c) are the images with 2%, 4%, and 6% of Salt and Pepper noise. (d), (e), and (f) are the images with Gaussian variance of 0.5%, 1%, and 1.5%.

WCE images are sometimes vulnerable to the noise contamination such as Gaussian noise or Salt and Pepper noise. To evaluate the performance of the proposed saliency map, we add three levels of Gaussian noise and Salt and Pepper noise in the WCE image in Figure 8.

**Figure 9:**
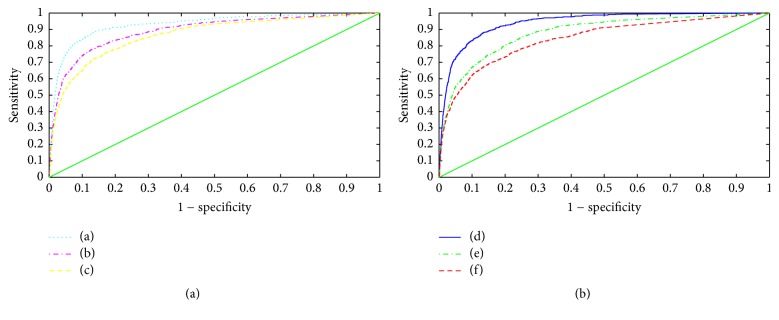
(a) is the performance of the fused saliency map with the images in Figure 8 with Salt and Pepper noise and (b) is on the images in Figure 8 with Gaussian noise.

From Figure 9 and Table 3, we can find that the accuracy of the proposed saliency map decreases as the level of noise increases, but the accuracy is still in expected range.

### 4.4. Run-Time Evaluation

The computation time of the fused saliency map and the contrast algorithm [17] is listed in Table 4. The algorithms are run on the MATLAB R2015b, with Intel Core i5 CPU at 2.4 GHz. Compared with [17], the proposed algorithm reduces the algorithm complexity of color saliency map and extracts three saliency maps in parallel. Table 4 shows that both algorithms are very fast and the proposed one has a minor advantage.

## 5. Conclusion

In this paper, a novel saliency map is proposed for bleeding localization on the WCE diagnosis. Unlike existing methods that use color features as the dominant criterion, we combine edge, intensity, and color information in visual saliency scheme. The results demonstrated that both the edge saliency map based on phase congruency and the intensity map based on luminance histogram dominate the saliency detection performance. Fused saliency map can detect the bleeding in WCE images with the average accuracy of 98.97%. Future work involves segmenting bleeding region with precise contours at a high specificity.

## Figures and Tables

**Table 1:** Performance comparison of the different saliency maps.

	Accuracy (%)	Sensitivity (%)	Specificity (%)
Edge	0.6372	**0.9878**	0.6317
Intensity	0.9684	0.8672	0.9736
Color	0.9496	0.7143	0.9615
Edge + intensity	0.9769	0.9329	0.9778
Edge + color	0.9635	0.8645	0.9774
Intensity + color	0.9779	0.8455	**0.9919**
[17]	0.9371	0.8958	0.9371
Fused	**0.9897**	0.9407	0.9915

**Table 2:** Performance comparison of the fused saliency maps in the different color space.

	RGB	HSI	HSV
Sensitivity (%)	0.9407	0.9420	0.9393
Specificity (%)	**0.9915**	0.9755	0.9804
Accuracy (%)	**0.9897**	0.9695	0.9744

**Table 3:** Comparison of accuracy parameters of the fused saliency map with different level noise.

Level	Salt and Pepper (%)	Gaussian (%)
1	98.94	99
2	98.51	98.44
3	98.16	98.15

**Table 4:** Computation time comparison.

	The fused saliency map	[17]
Time/per (s)	0.0748	0.0855
